# A scoping review of the present condition of organ donation among Chinese citizens and its influencing factors

**DOI:** 10.3389/fpubh.2026.1915223

**Published:** 2026-09-30

**Authors:** Xianyong Liu, Di Sun, MioLeng Au, Wangxuan Zhou

**Affiliations:** 1 Nursing School of Hunan Normal University, Changsha, Hunan, China; 2 Kiang Wu Nursing College of Macau, Cotai, Macao SAR, China; 3 The First People’s Hospital of Huai’an, Huai’an, Jiangsu, China

**Keywords:** Chinese citizens, influencing factors, organ donation, scoping review, willingness to donate

## Abstract

**Objective:**

This study aims to systematically analyse the current status of post-mortem organ donation among Chinese citizens and its influencing factors through a scoping review. It also seeks to identify the characteristics and gaps in existing research and provide evidence to inform policy directions for strengthening China’s organ donation system and improving organ donation practices.

**Methods:**

Guided by the scoping review methodology framework proposed by Arksey and O’Malley, a systematic search and screening of Chinese and English databases—including PubMed, Web of Science, Embase, CNKI, Wanfang, and VIP—was conducted to collect relevant articles on post-mortem organ donation. The search period spanned from the inception of the databases to 1 May 2025. Thematic analysis was employed to categorise and analyse the literature data.

**Results:**

A total of 19 articles were ultimately included, from which six themes influencing organ donation were identified: socio-demographics, social environment, legal systems, healthcare systems, individual cognition and attitudes, and public awareness and education.

**Conclusion:**

Compared with other countries, the willingness to donate organs after death among Chinese citizens is relatively low, and the overall donation rate urgently needs to be improved. Future research should focus on identifying key factors influencing donation willingness, developing standardised assessment tools, and strengthening qualitative research to gain a deeper understanding of the decision-making processes of potential donors. On this basis, targeted assessment and intervention strategies should be formulated to effectively increase organ donation rates and optimise the development of the system.

**Systematic review registration:**

https://doi.org/10.17605/OSF.IO/7G5RM.

## 1 Introduction

Organ donation and transplantation represent the optimal clinical solution for patients with end-stage organ failure, offering not only significant therapeutic benefits but also exceptional cost-effectiveness. Since the mid-20th century, transplantation techniques have advanced rapidly within modern medicine, becoming one of the most remarkable medical achievements of the 20th century (1, 2). However, the global imbalance between organ supply and demand remains severe. According to statistics from the World Health Organisation (WHO), more than 1.5 million people worldwide await organ transplants each year, yet only approximately 130,000 are able to receive a transplant (3). In China, as of 1 May 2025, the cumulative number of registered voluntary organ donors exceeded 7.07 million, with 58,906 post-mortem organ donations completed. Currently, China ranks first in Asia and second globally in terms of organ donation and transplantation volumes (4), Nevertheless, over 140,000 patients in China remain on the brink of life and death whilst awaiting organ transplants, with only approximately 20,000 ultimately receiving a new lease of life. The supply-to-demand ratio stands at 1:7.35; compared with developed countries, China’s donation rate per million population (approximately 4–5 PMP) remains less than one-tenth of that in Spain (5, 6). This disparity indicates that China’s organ donation system still faces severe challenges, with a large number of patients unable to receive timely treatment, necessitating urgent in-depth research and improvements. Therefore, a systematic analysis of the factors influencing post-mortem organ donation is of great significance. To date, domestic and international research has examined the factors influencing organ donation from multiple perspectives. Internationally, these factors fall into four major categories: policy and institutional frameworks, medical infrastructure, socioeconomic and demographic structures, and societal notions of altruism. Among these, policy and institutional frameworks have the most significant impact: compared to the “opt-in” system, the “opt-out” system can substantially increase organ donation rates (7). Compared to other countries, the willingness to donate in China is significantly constrained by local traditional culture, which manifests in three practical barriers: the traditional concept of bodily integrity, the issue of family vetoes resulting from family-centered decision-making, and the funeral tradition of “honoring the deceased and honoring one’s ancestors,” which leads to organ donation being viewed as disrupting the integrity of rituals and labeled as “disrespectful to parents” or “inauspicious” (8) Previous domestic studies have categorized influencing factors into three major categories: individual internal factors, family environmental factors, and external social environmental factors (9). However, previous studies have largely focused on a single dimension or specific populations, lacking integration and synthesis. As a systematic literature review method, a scoping review can comprehensively examine the breadth and depth of a research field, summarise existing research findings, identify research gaps, and provide a higher level of evidence than traditional reviews (10–12). Guided by the scoping review methodology, this study systematically collated and analysed relevant literature on the factors influencing post-mortem organ donation among Chinese citizens. It aims to elucidate the current research landscape, identify key influencing factors and research gaps, and provide evidence to inform policy directions for strengthening China’s organ donation system and improving organ donation practices. Furthermore, the findings may provide a basis for future research and policy development.

## 2 Materials and methods

This study was guided by the scoping review methodology framework proposed by Arksey and O’Malley (13), comprising the following five steps: (1) defining the research question; (2) searching for relevant studies; (3) screening studies; (4) extracting data; and (5) organising, summarising and reporting the results. The review was conducted and reported in accordance with the Preferred Reporting Items for Systematic reviews and Meta-Analyses extension for Scoping Reviews (PRISMA-ScR) checklist (14). The completed PRISMA-ScR checklist is provided as Supplementary file 1. A brief methodological appraisal was conducted using the AXIS tool for cross-sectional studies and the JBI critical appraisal tool for the retrospective cohort study (Supplementary Table S1). The appraisal was used to support interpretation of the findings rather than as an exclusion criterion. The appraisal was used to support interpretation of the findings rather than as an exclusion criterion. This study protocol has been registered with the Open Science Framework (OSF) under the registration number https://doi.org/10.17605/OSF.IO/7G5RM.

### 2.1 Defining the research question

In accordance with the Participants, Concept, Context (PCC) principle (15), this study defined the following research subjects and scope: the research subjects are Chinese citizens; the research concept is organ donation; and the research context is the current status of post-mortem organ donation among Chinese citizens and its influencing factors. The following research questions were ultimately formulated: ① What is the current status of post-mortem organ donation among Chinese citizens? ② What are the key factors influencing post-mortem organ donation among Chinese citizens? ③ What are the strategies for organ donation both domestically and internationally?

### 2.2 Literature review

This study conducted a systematic search of Chinese and English databases, including PubMed, Web of Science, Embase, CNKI, Wanfang, and VIP. The search strategy combined subject headings, free-text terms, and Boolean operators, supplemented by citation tracing. The detailed search strategies for each database are provided in Supplementary Table S1. The search time frame spanned from the inception of each database to 1 May 2025. Search terms included: “Tissue and Organ Procurement ‘,’ organ procurement system* ‘OR’ tissue procurement* ‘OR’ required request* ‘OR’ donor card*” ‘OR’ tissue donation* ‘OR’ organ donation* ‘OR’ Organ Shortage. Taking PubMed as an example, the specific search strategy is shown in Figure 1.

**Figure 1:**
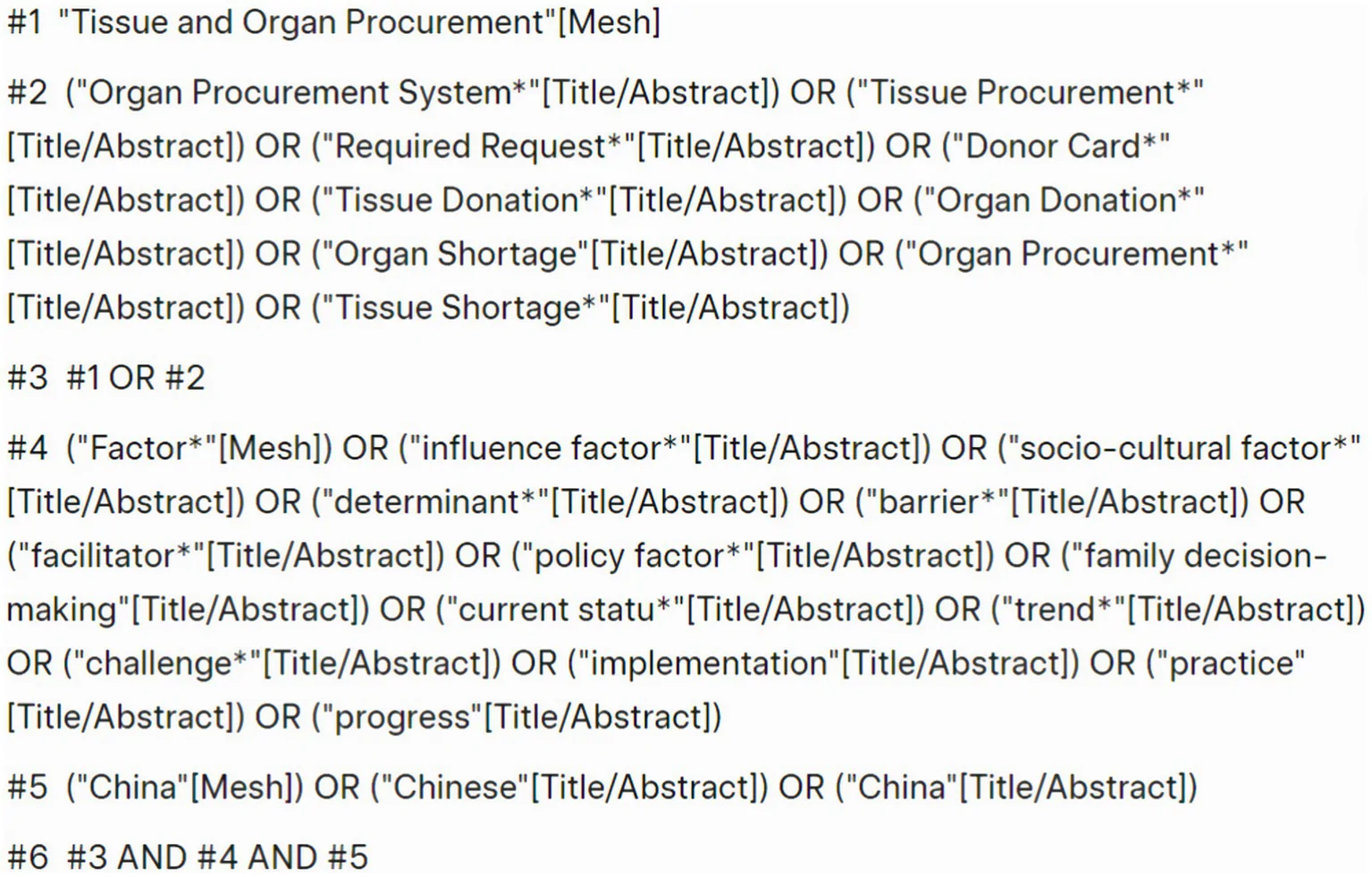
Search strategies of PubMed.

### 2.3 Literature screening

Inclusion criteria: ① The study subjects were Chinese citizens; ② The research topic concerned the current status of organ donation and its influencing factors; ③ The study design was quantitative (including interventional, cross-sectional and longitudinal studies), qualitative or mixed-methods; ④ Primary literature; ⑤ Language: Chinese or English; ⑥ Where studies targeted the same population, priority was given to those with the largest sample size and highest quality; ⑦ Original research. Exclusion criteria: ① Literature for which the full text could not be obtained; ② Duplicate publications; ③ Literature with small sample sizes from the same region; ④ Literature investigating only the willingness to donate organs from living donors.

### 2.4 Literature screening and data extraction

After importing the retrieved literature into EndNote 21 software to remove duplicates, two postgraduate students trained in evidence-based nursing independently read the literature and extracted information, cross-checking their results. Following an initial screening, full texts were read to conduct a secondary screening. In cases of disagreement, a third researcher was invited to participate in the discussion to jointly determine whether a study should be included. The included studies were analysed and summarised using thematic analysis (16). The researchers independently extracted information from the included studies using standardised forms, covering: first author, year of publication, study region, study type, sample size, and influencing factors. The influencing factors comprised 33 specific factors across 6 themes:

(1) Sociodemographic factors: age, gender, educational attainment, marital status, place of residence, occupation, economic status, political affiliation, family structure, cause of death, ethnicity. (2) Socio-environmental factors: traditional beliefs, religious beliefs, public opinion, family attitudes, social support. (3) Legal and policy factors: legal safeguards, policy incentives, financial compensation. (4) Healthcare system factors: trust in healthcare, organ allocation, understanding of donation procedures, post-donation arrangements; (5) Individual cognitive and attitudinal factors: personal beliefs, level of understanding regarding organ donation, psychological state, concerns regarding dignity, medical background, altruistic disposition, health literacy; (6) Publicity and education factors: intensity of publicity, participation in public welfare initiatives, personal experience of donation.

## 3 Results

### 3.1 Results of the literature screening

An initial search of the databases yielded 2,926 records, and an additional 4 records were identified through citation tracking, resulting in a total of 2,930 articles. After removal of 379 duplicate records using EndNote 21 software, 2,551 articles remained. Following title and abstract screening, 2,469 records were excluded and 82 records were assessed in full text. Of these, 63 records were excluded, including 13 for inappropriate study design, 15 for lack of relevance to factors influencing organ donation, 18 for an inappropriate study population, and 17 for a small sample size. Each excluded study was assigned to one primary reason for exclusion. When a study met more than one exclusion criterion, only one primary reason was recorded to ensure that each study was counted once and to avoid double-counting across exclusion categories. Finally, 19 studies were included in the review.

The characteristics of the included articles are shown in Table 1.

**Table 1:** Basic characteristics of the included studies (a: cross-sectional study; b: retrospective cohort study).

Author (year)	Study area	Study population	Sample size (*n*)	Factors
Zixiang et al. (17), 2018	Shandong	The public	2511^a^	Age, educational background, traditional beliefs, legal protections, personal views, public opinion
Peixuan et al. (18), 2019	Zhejiang	The public	617^a^	Occupation, altruistic personality traits, level of awareness regarding organ donation, and the extent of public awareness campaigns
Lina et al. (19), 2019	Shaanxi	The public	1480^b^	Age, family structure, economic status, educational attainment, marital status, religious beliefs, place of residence, occupation, cause of death
Ling et al. (20), 2019	Hubei	The public	1192^a^	Age, personal beliefs, prior donation experience, and level of awareness regarding organ donation
Jing et al. (21), 2019	Guizhou	ICU medical staff	926^a^	Gender, place of residence, educational attainment, and awareness of organ donation
Lu Yiqiao et al. (22), 2020	Shandong, Zhejiang, Hunan	The public	1886^a^	Family attitudes, personal beliefs, level of awareness regarding organ donation, organ allocation, and policy incentives
Wei et al. (23), 2020	Zhejiang	The public	1886^a^	Age, educational background, family attitudes, organ allocation, personal beliefs, trust in the medical system
Xiang Zhang et al. (24), 2020	Yunnan	ethnic minorities	1680^a^	Occupation, religious beliefs, economic status, educational attainment, barriers related to the donation process, policy incentives
Jeremy YC Teoh et al. (25), 2020	Hong Kong	Hong Kong residents	1000^a^	Personal beliefs, traditional views, family attitudes, trust in the healthcare system, participation in public welfare initiatives, the extent of public awareness campaigns, and barriers related to the donation process
Ning et al. (26), 2020	Heilongjiang	college student	975^a^	Traditional beliefs, trust in the medical system, the extent of public awareness campaigns, financial compensation, and obstacles related to the donation process
Lijuan et al. (27), 2021	Nationwide	The public	1580^a^	Age, educational attainment, social support, awareness of organ donation, policy incentives, family attitudes, traditional beliefs, financial compensation, legal protections, public opinion
Yang Xin et al. (28), 2021	Shenyang	The public	8942^a^	Age, gender, educational attainment, marital status, economic status, occupation, political affiliation, religious beliefs
Zhang et al. (29), 2022	Nationwide	The public	11031^a^	Gender, place of residence, mental health, social support, health literacy, family structure, religious beliefs
Fan et al. (30), 2022	Zhejiang, Henan, Shaanxi	The public	4274^a^	Place of residence, marital status, gender differences, educational attainment, traditional beliefs, level of awareness regarding organ donation, and barriers related to the donation process
Guo, et al. (31), 2022	Nationwide	The public	4565^a^	Gender, age, religious beliefs, place of residence, educational attainment, awareness of organ donation, and social support
Minghao et al. (32), 2022	Shandong	college student	988^a^	Gender, family attitudes, medical background, level of awareness regarding organ donation, and barriers related to the donation process
Yang et al. (33), 2023	Guangxi	Faculty and students at medical schools	638^a^	Traditional beliefs, public awareness of organ donation, post-donation arrangements, trust in the medical system, concerns about dignity, and financial compensation
Qiu Qingfan et al. (34), 2024	Guangxi	college student	974^a^	Traditional beliefs, public awareness of organ donation, post-donation arrangements, trust in the medical system, barriers related to the donation process, policy incentives, economic status
Fuli et al. (35), 2024	Xinjiang	The public	1031^a^	Age, educational attainment, personal beliefs, civic engagement, history of charitable giving, awareness of organ donation, barriers to organ donation

### 3.2 Basic characteristics of the literature

A total of 19 studies were included in this review (17–35), These comprised 15 Chinese-language studies and 4 English-language studies. The research covered the whole of China. The study designs included 18 cross-sectional studies and 1 retrospective cohort study, with sample sizes ranging from 617 to 11,031 (18, 29) cases. The basic characteristics of the included studies are shown in Figure 2.

**Figure 2:**
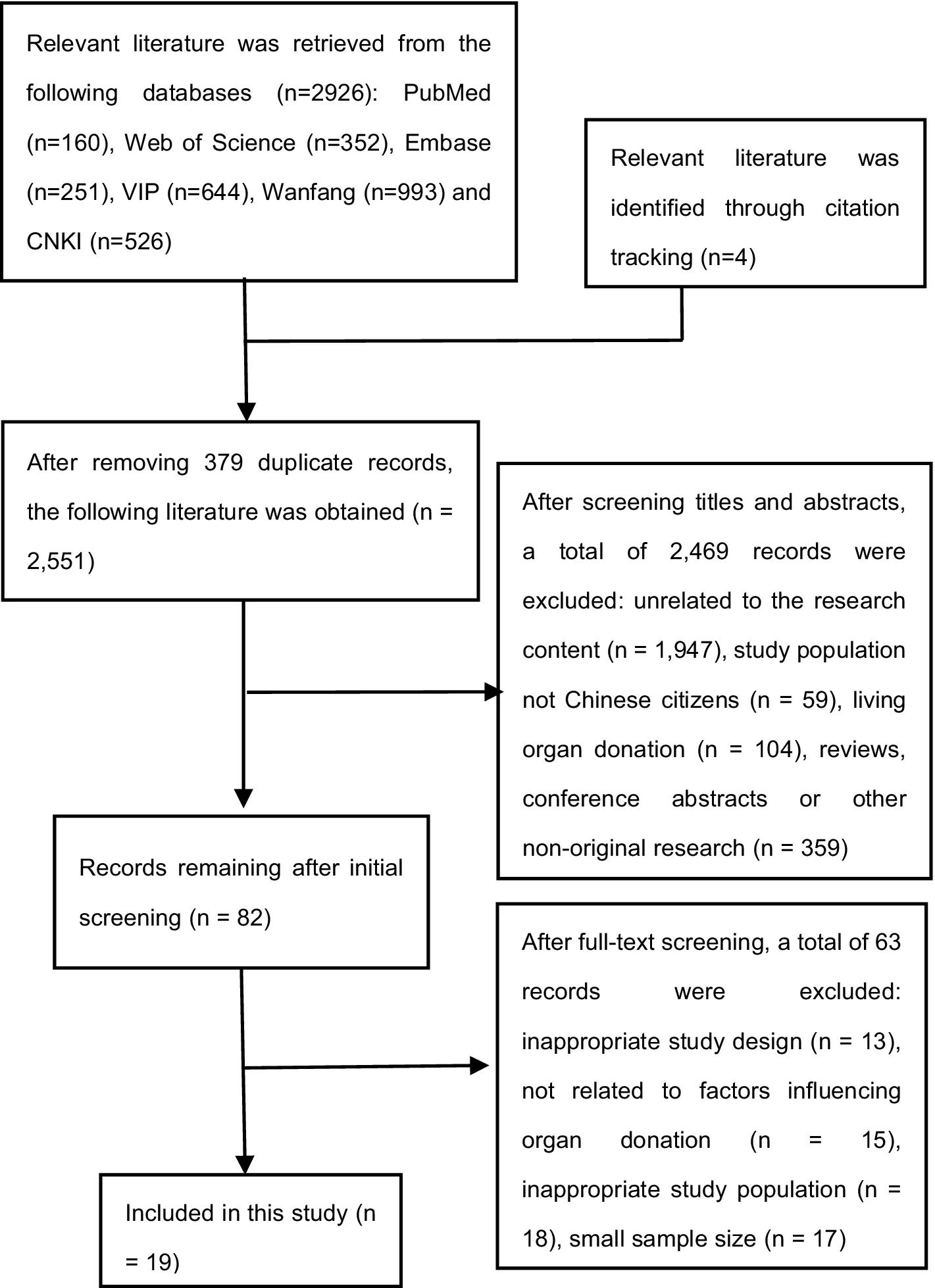
Characteristics of the included studies.

### 3.3 Current status of research on post-mortem organ donation among Chinese citizens

Of the 19 included studies, 17 reported the rate of willingness to donate organs after death among Chinese citizens, ranging from 1.9% to 69.9% (24, 28). None of the primary studies reported 95% confidence intervals for these estimates. The methodological appraisal identified some limitations in sampling methods, study populations, and outcome reporting across the included studies. These limitations should be considered when interpreting these reported values. Among these, the willingness to donate among ethnic minorities is significantly lower than that of the Han Chinese. For example, the willingness rate among 420 members of the Hani ethnic group was 1.9%, whilst that of other ethnic minorities—such as the Bai (2.0%), Yi (2.1%) and Dai (2.3%)—was also lower. In contrast, the rate among the Han Chinese, who are influenced by Confucian culture, stood at 10.9%. Religious beliefs and economic circumstances are considered to be the primary influencing factors. However, the willingness to donate one’s body among residents of Shenyang stands at 69.9 per cent, far exceeding the national average. This is largely attributable to intensive information dissemination via the mass media, which has raised awareness of organ donation among local residents to 91.8 per cent, significantly mitigating the inhibitory effect of traditional beliefs and concerns about risks on donation decisions.

### 3.4 Factors associated with organ donation following a person’s death

This study identified six themes of factors associated with post-mortem organ donation, as detailed below. Most studies examining these factors used a cross-sectional design; therefore, the findings below describe observed associations rather than causal relationships.

#### 3.4.1 Sociodemographic factors

Studies reported that younger age (17, 20, 23, 27, 28, 31), female gender (21, 28–32), higher educational attainment (17, 21, 23, 24, 27, 28, 30, 31, 34), being married (16, 29), and urban residence compared with rural residence (24, 29–31) were associated with greater willingness to donate organs. A larger number of immediate family members was negatively associated with willingness to donate (19, 29). Among the reported causes of death, patients with traumatic brain injury or cerebral haemorrhage showed greater willingness to donate organs (19). Furthermore, lower income levels were associated with greater willingness to donate in some studies (19, 28).

#### 3.4.2 Sociocultural factors

Traditional beliefs such as “resting in peace” and “preserving the body’s integrity after death” were consistently associated with lower willingness to donate organs (17, 25–27, 30, 33, 34). The family occupies a central position in Chinese culture, and the attitudes of family members were strongly associated with donation decisions (22, 23, 25, 32). Higher levels of social support were associated with greater willingness to donate (27, 29, 31). However, negative public discourse surrounding organ donation in China was associated with lower willingness to donate (17, 27).

#### 3.4.3 Legal and institutional factors

A well-established organ donation system, sound laws and regulations, and effective interdepartmental coordination and communication were associated with greater willingness to donate (16, 27). Incentive policies, such as conferring social recognition, providing end-of-life care, and offering government-sponsored benefits, were positively associated with public willingness to donate (22, 24, 27, 34). Studies have found that Han Chinese tend to prefer non-monetary incentives (such as certificates of honor), while ethnic minorities tend to prefer financial compensation (24). Furthermore, moderate financial compensation was associated with greater willingness to donate organs (26, 27, 33).

#### 3.4.4 Factors related to the healthcare system

Lower levels of trust in the healthcare system were associated with lower willingness to donate organs. The reported concerns involved trust in administrative bodies, donor registration systems, organ allocation systems, hospitals, physicians, and organ donation coordinators (22, 23, 25, 26, 33, 34). In addition,limited understanding of the donation process and its complexity was associated with lower willingness to donate (24–26, 32, 34). Concerns about issues such as inadequate improvement in recipients’ health outcomes and inappropriate post-donation procedures were also associated with lower willingness to donate (33, 34).

#### 3.4.5 Individual cognitive and attitudinal factors

The associations between individual beliefs and organ donation willingness varied across studies (17, 20, 22, 23, 25, 35). Across the included cross-sectional studies, greater knowledge of organ donation was positively associated with willingness to donate (17, 18, 20–23, 25, 30, 32, 34, 35). In a cross-sectional study, Zhang et al. (29) reported a positive association between mild-to-moderate anxiety and willingness to donate. Additionally, a cross-sectional study found that medical students had a higher willingness to donate organs than non-medical students (32).

#### 3.4.6 Public awareness and education factors

Limited public awareness of organ donation registration procedures was associated with lower understanding and motivation to donate (18, 25, 26). Participation in public welfare activities was positively associated with willingness to donate organs (25, 35). At the same time, members of the public who have personal experience with organ donation or whose relatives or friends have donated organs reported higher willingness to donate (20, 35).

## 4 Discussion

### 4.1 Analysis of the current state of research on post-mortem organ donation among Chinese citizens

This study included 19 relevant literature sources. The results indicate significant heterogeneity in the rates of willingness to donate organs after death among Chinese citizens and the factors influencing them. The primary reasons for these differences include: (1) Heterogeneity of study subjects: Diversity across different regions and populations leads to marked differences in public awareness and attitudes toward organ donation. (2) Methodological differences: The assessment tools and question designs used across studies were inconsistent, affecting the comparability and consistency of the data. (3) Insufficient sample representativeness: Sample sizes varied widely; some studies had small sample sizes, making it difficult to adequately represent the target population. (4) Limited research depth: Most studies rely on questionnaire surveys and lack in-depth communication with respondents, making it difficult to fully reveal their true attitudes and motivations. Based on the above analysis, future research should focus on developing standardized assessment tools and conducting large-scale, multicenter studies nationwide, while incorporating qualitative interviews to more deeply explore the underlying factors influencing willingness to donate organs, thereby providing a reliable basis for policy formulation and practice improvement.

### 4.2 Analysis of factors associated with organ donation

#### 4.2.1 Sociodemographic factors

Awareness and attitudes toward organ donation vary across different demographic groups, and differences in willingness to donate have been reported across these groups. The relationship between age, gender, educational attainment, marital status, place of residence, and willingness to donate organs remains a subject of debate. Studies reported that younger individuals had greater willingness to donate organs than older adults (17, 20, 23, 27, 28, 31). Female gender was associated with greater willingness to donate.

organs (17, 21, 23, 24, 27, 28, 30, 31, 35). Individuals with higher levels of education were more willing to donate organs (17, 21, 23, 24, 27, 28, 30, 31, 35). Urban residents were more willing to donate than rural residents (29) A study by Mengjun Zeng et al. (9) similarly reported that younger people, women, those with higher levels of education, and urban residents had greater willingness to donate. However, the number of immediate family members was negatively associated with willingness to donate (19, 29). Analyzing the reasons, people with children (especially those in families with multiple children) may be more inclined to follow this tradition to avoid being perceived as “disrespectful to their parents.” Furthermore, family members of patients with traumatic brain injury or cerebral haemorrhage reported greater willingness to donate (19). Similarly, a large Italian registry-based study involving 10,254 potential donors found that traumatic brain injury was associated with a lower likelihood of family refusal of organ donation (36). Although this study was conducted in a different cultural and healthcare context, its findings provide an international comparison for the observed association between traumatic brain injury and family decisions regarding organ donation. These findings suggest that organ donation education and outreach may benefit from considering differences across population groups.

#### 4.2.2 Medical and social environmental factors

Trust in the healthcare system was an important factor associated with willingness to donate organs. Studies indicate that only 25.2% of the Chinese public trusts the healthcare system (37). Lower trust in the healthcare system was associated with lower willingness to donate. Previous studies have discussed historical medical incidents and information asymmetry as possible explanations for this concern (25, 26). Specific concerns include: 57.71% of college students worry that medical personnel may declare donors dead prematurely to obtain organs; 49.81% do not trust the professional ethics of organ donation coordinators; and 25.93% are concerned that the deceased’s body will not be treated with respect (26). These findings suggest that improving transparency in organ allocation, strengthening doctor–patient communication, and providing accessible public information may be relevant areas for future policy and practice. Furthermore, the public’s lack of understanding regarding organ donation procedures and the complexity of the process was associated with lower willingness to donate (24–26, 32, 34). These findings highlight the importance of developing an efficient, accessible, and patient-centred donation system. These findings highlight the potential value of improving public education and simplifying donation procedures. In traditional Chinese culture, filial piety is deeply ingrained as a core value. The traditional belief that “one’s body and hair are gifts from one’s parents, and one must not harm them” (38) was associated with perceptions of organ donation as potentially violating bodily integrity and conflicting with the principles of filial piety (17, 25–27, 30, 33, 34). Culturally sensitive education may provide a useful approach to addressing these concerns and improving public understanding of organ donation. In Chinese culture, family opinions are closely associated with individual decision-making regarding organ donation (22, 23, 25, 32). Influenced by Confucian traditions, family opinions often serve as a key determinant of donation willingness. On the other hand, higher levels of social support were associated with greater willingness to donate (17, 29, 31). Such support—especially when it offers emotional care and a sense of social honor—may be conducive to greater public participation and could help alleviate the psychological burden on donors’ families. In the future, the social support system should be further improved to provide a more solid practical foundation for organ donation.

#### 4.2.3 Legal system factors

China’s legal framework for organ transplantation has developed progressively over the past several decades. Since the first kidney transplant in 1960, the system has progressed through various stages, including exploratory local legislation, the 2007 ban on organ trafficking, the 2010 pilot scheme for voluntary citizen donation, and the 2015 complete cessation of the use of organs from executed prisoners, culminating in the new regulations coming into force on 1 May 2024. However, the legal definition of brain death remains unclear, and issues such as the criteria for determination and transparency in allocation continue to provoke ethical controversy (39, 40). Surveys indicate that only 13.42% of the population understand the concept of brain death (27), whilst approximately 61% consider the fairness and transparency of the organ allocation system (22). Currently, transplant organs are allocated centrally via a national computerised system; however, cross-regional allocation has also been discussed in relation to challenges such as insufficient information sharing and the uneven distribution of medical resources and technology. Gaps in the existing legal and institutional framework have been discussed as potential barriers to organ donation. For example, some deceased individuals who wished to donate organs during their lifetime face opposition from their families, whilst hospitals are reluctant to accept voluntary donations due to a lack of legal safeguards or to avoid risks. Furthermore, incentives such as financial compensation and social recognition were positively associated with willingness to donate (22, 24, 27, 34), Reported forms of incentives included funeral subsidies, waivers of medical fees, and increased health insurance reimbursement rates (26, 27, 33), These findings should be considered alongside the ethical concerns surrounding financial incentives for organ donation. China has tentatively established a policy framework for organ donation centred on non-monetary incentives; however, this remains at the stage of institutional refinement and local pilot schemes, and a unified, systematic national legal framework has yet to be established. Caution is needed when considering the applicability of Spain’s presumed-consent (opt-out) model to China, given differences in cultural, family, and legal contexts. China should build upon its national circumstances and explore a “Chinese-style organ donation” approach that aligns with its social, cultural and ethical context.

#### 4.2.4 Individual perceptions and public awareness factors

Individual perceptions were associated with willingness to donate organs. Surveys indicate that 38.8% of people consider whether they accept the concept of organ donation as their primary factor (22). Traditional beliefs, religious convictions, and individuals’ acceptance of the value of organ donation were associated with donation decision-making (17, 20, 22, 23, 25, 35). Although most citizens have a basic understanding of organ donation, only 20.4% are aware of who holds the decision-making authority regarding post-mortem donation (18). Awareness of the possibility of revoking donation registration was associated with greater willingness to donate (17). Studies indicate that the level of understanding regarding organ donation is positively correlated with the willingness to donate (17, 18, 20–23, 25, 30–32, 34, 35). Trust, clarity regarding ethical issues, confidence in the legal framework, sense of responsibility, and psychological concerns were identified as relevant considerations in donation decision-making. However, the public lacks sufficient understanding of the concept of “brain death,” the donation process, and the age eligibility for donation, and these gaps are often accompanied by concerns about organ abuse or the violation of the dignity of the deceased. Furthermore, only 16.3% of residents are aware that they can register for organ donation via Alipay (17). These findings highlight the importance of public education and outreach in improving understanding of organ donation. Psychological state was also associated with willingness to donate. In a cross-sectional study, mild-to-moderate anxiety, moderate stress, and severe stress were positively associated with willingness to donate (29). This may be because anxiety prompts individuals to reflect on the meaning of life and stimulates altruistic motives. Medical students were reported to have greater willingness to donate than non-medical students (26, 32).

This finding highlights the relevance of educational background to attitudes toward organ donation. Given that college students are a young and highly receptive demographic, it is recommended that schools offer courses on organ donation, provide education on perspectives on life and death, and organize in-person activities to help students understand the significance of preserving life and overcome the psychological barriers associated with death and bodily integrity that stem from traditional culture. Spain has been widely recognised as an international example of a well-developed organ donation system, with 52.6 deceased donors per million population reported in (41). Simon Streit et al. (42) identified three key components of Spain’s organ donation system: building trust through media collaboration and public education, enhancing efficiency through governance structures and a network of coordinators, and supporting allocation through transparent laws and technology. Rees et al. (43) noted through qualitative analysis that Spain’s simpler, locally adapted consent documents, shorter family decision times, stronger legal protection for prior decisions, and supportive language collectively support higher consent rates. However, differences in family values, cultural context, and legal systems should be considered when assessing the applicability of Spain’s “opt-out” model to China. Taking into account its national context, China may consider public awareness strategies such as mainstream media campaigns highlighting inspiring stories, 24-h hotlines, and National Donation Days. Face-to-face booth activities in Hong Kong also offer valuable insights. In one study, 47.7% of registered donors completed their registration through such events (26). This finding suggests that face-to-face interaction may be a useful approach for facilitating donor registration. In the future, China could consider promotional strategies such as the “Chengguang Model,” which may provide a useful reference for future organ donation awareness campaigns (17).

Overall, the findings indicate that willingness to donate organs among Chinese citizens is associated with multiple factors, including sociodemographic characteristics, cultural and family values, trust in the healthcare system, legal and institutional factors, individual knowledge and perceptions, and public awareness. These factors were reported across different population groups and study settings, suggesting that organ donation decision-making is multidimensional. The findings highlight several areas for future policy and practice, including improving public understanding of organ donation, strengthening institutional trust and transparency, and developing supportive legal and social environments. Given the heterogeneity of the existing evidence, particularly in reported willingness to donate, further research using standardised measures and diverse study designs is warranted.

### 4.3 Limitations

This study has certain limitations: (1) Research Design: The included literature consists primarily of cross-sectional studies, making it difficult to explore the subjects’ feelings, attitudes, motivations, and related needs in depth, thereby limiting the exploration of underlying psychological and behavioral factors; (2) Measurement Tools: The lack of consistency in the questionnaires and scales used across different studies resulted in high data heterogeneity, thereby affecting the comparability and overall reliability of the results; (3) Sample Representativeness: Most studies were limited to specific regions or schools, with small sample sizes, failing to adequately reflect the general situation nationwide and thus lacking sufficient representativeness. To overcome these limitations, future research is advised to adopt standardized scales with higher reliability and validity, conduct multicenter, large-scale surveys, and incorporate qualitative interviews with research participants and their families to more accurately identify the needs of potential donors. These improvements will help enhance the breadth and reliability of research findings, providing more valuable reference data for optimizing organ donation rates.

## 5 Summary

Through a scoping review of 19 studies, this research provides a systematic summary of the key factors influencing postmortem organ donation among Chinese citizens. The study identified several factors influencing post-mortem organ donation among Chinese citizens, including legal and institutional factors, social environmental factors, healthcare system factors, and individual cognitive factors. These factors can serve as a reference for optimizing public attitudes toward organ donation and designing intervention strategies. However, the “Chinese-style” organ donation system is still in its early stages of development and remains far from achieving the goals of maturity and internationalization. To strengthen China’s organ donation system, sustained efforts are needed in the following areas: At the national level: improve the policy and regulatory framework to provide institutional safeguards for organ donation; at the government level: strengthen local enforcement and regulatory effectiveness; at the societal level: promote a shift in cultural attitudes and increase public participation; at the hospital level: enhance professional capabilities and technical standards; at the individual level: support voluntary donation and family involvement Furthermore, organ donation involves profound issues related to individual subjective values and humanitarian beliefs; quantitative data alone cannot fully reveal its underlying motivations. Therefore, future research should further strengthen qualitative research methods to deeply analyze the intentions and needs of potential donors, while also intensifying research on xenotransplantation to provide new insights and approaches for addressing the organ shortage.

## Data Availability

The original contributions presented in the study are included in the article/Supplementary material, further inquiries can be directed to the corresponding author.
